# Experimentation and Hospitals

**Published:** 1899-03-11

**Authors:** 


### The Hospital

A Journal of

The Medical Sciences and Hospital Administration.

Vol. XXV.—No. 650.] EDITED BY SIR HENRY BURDETT, K.C.B., AND SOLOMON C. SMITH, M.D., M.R.C.P. [March 11, 1899.

### EXPERIMENTATION AND HOSPITALS.

The enterprising people who are concerned in the management of the society or societies, for we believe there is more than one, which have been established for the purpose of hindering the advancement of medicine by research, and who find their account in describing every kind of experiment as "Vivisection," with a very large V, have of late developed a new and especially hurtful form of activity, which is carried on by assiduous circularising of the supporters of hospitals, in order to induce them not to contribute to the support of institutions which have among the members of their staffs gentlemen who are holders of licences for experimentation from the Home Secretary. It is not quite apparent with what object this course is pursued, because, as a rule, if not invariably, the experimentation is absolutely unconnected with the hospital, is conducted in a different building, and is merely one of the means of study pursued by the holder of a hospital appointment, and pursued quite apart from his discharge of the duties which that appointment imposes upon him. Neither is it quite sure what good is to be obtained by endeavours to injure a hospital, or, in other words, to compel the closure of some of its beds, to diminish the number of sufferers who can be relieved by its proceedings, and to curtail the efficiency of its organisation. All these effects are necessarily produced by want of funds. We have heard it suggested that the real object is to induce the governing bodies of hospitals to exclude experimenters from their staffs, or, in other words, to compel hospital surgeons and physicians to obtain all new knowledge at second hand; to withdraw from experimenters all opportunities of themselves applying the results of their work; and to injure their prospects of professional advancement. We can hardly conceive it possible that so entirely mean and base a project as this could be avowedly entertained, even by the old women (of both sexes) to whom the societies are mainly indebted for financial support, and still less by the more or less respectable figureheads behind the shelter of whose names the routine of agitation is carried on. None the less, it seems to us to be incumbent upon the latter to exercise some control over their nominal subordinates and actual rulers, and to place some check upon conduct which is neither more nor less than a disgrace to humanity.

The question whether an experiment on an animal, even though it be attended by the infliction of some suffering, is justifiable on the plea that it will probably or certainly be the means of diminishing the sufferings of other animals or of mankind, is one on which, it is abundantly evident, there is room for a legitimate and even natural difference of opinion. The evil of the experiment on the one hand, and of the suffering which it is intended to relieve, on the other, will present themselves with different degrees of force to different intellects, to different imaginations, and to persons differently qualified by information to arrive at correct and unprejudiced conclusions. So much is an inevitable result of the constitution of human nature; although, be it observed, there are extreme cases as to which mankind would be practically unanimous. No sane person could object to inflicting a pin-prick upon a rabbit, if by such a course the experimenter obtained power to extinguish such a malady as the plague. No sane person would approve of subjecting a large number of animals to prolonged torture, in order to cure one child of a chilblain. All actual cases lie between such extremes; and a very large majority of people are prepared to admit that the infliction of suffering upon animals may sometimes be justified by the consequent gain. Those who hold this view are as much entitled to their opinions, and have as much right to be guided by them, as the most extreme anti-vivisectors themselves; while the justifiable or unjustifiable character of any individual experiment or course of experiments can only be determined by those who are complete masters of the state of existing knowledge on the point at issue, and of the purposes to which additions to this knowledge might be applied. We have no respect for the intellect of the anti-vivisector, but we admit that he is entitled to his opinions, and that he is fully within his rights in any fair endeavour to win other people over to his views. We do not admit that he is entitled to use systematic falsehood for this purpose; and the suggestion that he may properly endeavour to dry up the sources of charity to hospitals, or to inflict professional injury upon medical men who do not agree with him, seems to us to be a striking illustration of the inherent baseness and cruelty of some of the lower depths of human nature, even when these are partially disguised under a profession of benevolence.

